# “The strategies are the same, the problems may be different”: a qualitative study exploring the experiences of healthcare and service providers with medication therapy management for individuals with spinal cord injury/dysfunction

**DOI:** 10.1186/s12883-019-1550-9

**Published:** 2020-01-15

**Authors:** Sara J. T. Guilcher, Amanda C. Everall, Tejal Patel, Tanya L. Packer, Sander L. Hitzig, Stephanie R. Cimino, Aisha K. Lofters

**Affiliations:** 1 Leslie Dan Faculty of Pharmacy, University of Toronto, Toronto, Canada; 2 Rehabilitation Sciences Institute, Faculty of Medicine, University of Toronto, Toronto, Canada; 3 Institute of Health Policy, Management and Evaluation, University of Toronto, Toronto, Canada; 4 School of Pharmacy, University of Waterloo, Kitchener, Canada; 5 Department of Family Medicine, DeGroote School of Medicine, McMaster University, Hamilton, Canada; 6 Schools of Occupational Therapy and Health Administration, Faculty of Health, Dalhousie University, Halifax, Canada; 7 St. John’s Rehab Research Program, Sunnybrook Research Institute, Sunnybrook Health Sciences Centre, Toronto, Canada; 8 Department of Occupational Science and Occupational Therapy, Faculty of Medicine, University of Toronto, Toronto, Canada; 9 Women’s College Hospital, Family Practice Health Centre, Toronto, Canada

**Keywords:** Spinal cord injuries, Professional roles, Medication therapy management, Medication adherence, Polypharmacy, Patient preference, Attitude to health, Attitude of health personnel

## Abstract

**Background:**

Persons with spinal cord injury/dysfunction (SCI/D) often take multiple medications to treat their secondary complications and chronic conditions (multimorbidity). Multiple healthcare and service providers are often involved in care, which can result in increased risk of fragmentation of care. Optimal medication therapy management (MTM) is essential to ensure therapeutic benefit from medication regimens. However, little is known about the experiences of providers in supporting persons with SCI/D with MTM.

**Methods:**

Telephone interviews were conducted to explore healthcare and service providers’ experiences with MTM for persons with SCI/D. Participants were recruited through clinical organizations and researchers’ personal contacts. Participants were purposefully selected for diversity in profession and were required to be English speaking and to have provided care to at least one person with SCI/D. The qualitative interviews followed a semi-structured interview guide. Data display matrices were used in a constant comparative process for descriptive and interpretive analysis.

**Results:**

Thirty-two interviews were conducted from April to December 2018. Each profession had distinct views on their roles in facilitating MTM for persons with SCI/D, which aligned with their respective scopes of practice. Shared provider tasks included tailoring medications, providing education, and exploring medication alternatives. Most participants felt that the care they provided for persons with SCI/D was similar to the care that they provided to other patients, with some differences relating to the physical limitations and medical complexity associated with SCI/D. Five factors were identified that impacted participants’ abilities to provide MTM for persons with SCI/D: patient self-management skills, provider knowledge and confidence, provider-patient relationships, interprofessional collaboration, and provider funding models including the use of technology-supported consultations.

**Conclusion:**

While participants described commonalities in the barriers and enablers associated with providing MTM to persons with SCI/D and other populations, there were unique considerations identified. These SCI/D-specific considerations resulted in recommendations for improvements in MTM for this population. Future research should include perspectives from persons with SCI/D.

## Background

People with spinal cord injury or dysfunction (SCI/D) typically take multiple medications (also known as polypharmacy) to manage secondary health complications (e.g., spasticity, urinary tract infections, pressure sores, respiratory infections) and multimorbidity (e.g., heart disease and diabetes) [1–6]. The reported polypharmacy rates among persons with SCI/D vary between 31 to 87% [6–9]. Pharmacotherapeutic treatment plans for persons with SCI/D often include a combination of prescription and over-the-counter medications and natural health products. Moreover, multiple health professionals can be involved in overall care [10], increasing the risk for the fragmentation of care and conflicting advice on how to self-manage complex medication regimens and treatment schedules [11].

In the general population, multimorbidity and problematic polypharmacy are associated with significant functional decline, increased healthcare utilization, decreased quality of life, and premature death [12–15]. Similarly, poorly managed chronic conditions (e.g., chronic pain, fatigue, depression) may have substantial impact on re-integration into the workplace, healthcare utilization, overall health, and quality of life of persons with SCI/D [16, 17].

While polypharmacy and multimorbidity are major global public health concerns in the general population [18], there is minimal research specific to the SCI/D population. Little is known about the complexity of medication therapy management (MTM) for persons with SCI/D and associated multimorbidity. Ideal MTM can be defined as *“patient-centred care to optimize safe, effective and appropriate drug therapy*” [19], with the “care” being a collaboration between the patient and care team members [19]. The lack of research on optimal MTM for SCI/D is surprising given that most persons with SCI/D experience significant complications directly and indirectly related to their injuries [1–5].

While a small body of research identifies a high prevalence of polypharmacy among persons with SCI/D [6–9], there remains a lack of critical research to help understand the experiences of different healthcare and service providers with MTM for this population. Therefore, the main purpose of this study is to understand the experiences of healthcare and service providers with MTM for persons with SCI/D, as well as the barriers and enablers of optimal MTM.

## Methods

### Study design

This study used a qualitative methodology to explore the experiences of healthcare and service providers supporting MTM. It received Research Ethics Board approval from the University of Toronto (human research protocol #34063) and the University of Waterloo (ORE #22902).

### Participants

Participants were healthcare providers (e.g., family physicians, nurses, nurse practitioners, pharmacists, occupational therapists, physical therapists) and service providers (e.g., care coordinators) in Canada. Maximum variation among participants (e.g., provider type, gender, years of experience) was sought to ensure a wide range of experiences were captured [20].

### Recruitment and screening

Participants were recruited through numerous avenues: emails sent to individual providers in the researchers’ networks and through professional listservs, flyers posted in various healthcare establishments, a lunch and learn conducted with a primary care team, advertisements on social media, and snowball sampling [21] (i.e., participants shared study information within their networks). Interested individuals were screened for three inclusion criteria: 1) being a healthcare or service provider in Canada, 2) having provided care to at least one person with SCI/D, and 3) being English speaking. Those individuals who passed the screening were sent a letter with study information and the consent form to review. Participants were asked to return the signed consent form to the research team; however, when written consent was not possible, verbal consent was obtained.

### Data collection

Semi-structured interviews with all participants were conducted by telephone and were divided into two parts: a short intake survey followed by open-ended qualitative questions related to MTM. Participants were provided with a definition and examples of MTM, which included a wide range of services that optimize therapeutic outcomes for patients, such as health assessments, medication reviews, medication treatment plans, monitoring of medications and the efficacy and safety of therapy, education, and self-management for patients [22]. The brief demographic survey questions asked about clinical practice and experience working with persons with SCI/D. For the open-ended questions, providers were asked to describe their experiences and roles with providing MTM for persons with SCI/D, barriers and enablers for supporting their patients, and strategies to help their patients with medication management.

Answers to the intake survey portion of the interview were hand-written by the interviewers and the qualitative portion of all interviews were audio-recorded. The audio-recordings were transcribed verbatim for analysis.

### Data analysis

Qualitative data analysis involved an iterative constant comparative process, which included descriptive and interpretive analyses [23]. The analyses used an inductive approach to identify emerging concepts from the data [21] and followed components of the Qualitative Analysis Guide of Leuven [24], such as creating short narrative interview reports and applying a constant comparison approach with input from the team. Data analysis was conducted concurrently with data collection to allow for emerging concepts to be included in subsequent data collection. The interviews were conducted until data saturation (i.e., no new themes or ideas) was identified.

A coding framework was created by the core research team with feedback from the broader team in an iterative process. First, four initial transcripts were reviewed by core team members who met to discuss emerging concepts. From these concepts, a preliminary coding framework (code names, definitions, and examples) was developed. Two team members applied the preliminary coding framework to two new transcripts using NVivo 11 and reported back to the broader team about the coding process and the coding framework. The coding framework was revised until consensus among the broader team was reached. Once the coding framework was finalized, two team members coded two additional transcripts and inter-coder agreement was compared [25]. An agreement of 98.3% was established between these coders, who proceeded to code all remaining transcripts using the finalized coding framework. The research team continued to meet regularly to discuss the most relevant themes that were identified from the data.

The software programs NVivo 11 and Microsoft Excel 2016 were used to compare codes by provider type and to assist in identification of themes. Overall, data saturation, constant comparative analysis, trustworthiness, and validity checks provided assurance of data quality and rigor [25, 26]. Descriptive statistics (means, standard deviations, medians, interquartile ranges, percentages) were used to analyze the socio-demographic data.

## Results

### Description of participants

Thirty-two healthcare and service providers were interviewed between April and December 2018. Thirty-eight percent of participants were pharmacists (*n* = 12) and 31% were physicians (*n* = 10). The majority of participants were female (*n* = 20) and had a median of 11 years working experience (IQR 15.8, range 1 to 37) (See Table 1).

**Table 1:** Participant Characteristics (*n* = 32)

Characteristics (*n* = 32)	Total
Sex	
Male	12
Female	20
Occupation	
Pharmacists (e.g., Community; Hospital)	12
Physicians (e.g., Family Physicians; Specialists)	10
Rehabilitation Professionals (e.g., Occupational and Physical Therapists; Chiropractors; Registered Nurses; Case Coordinators)	10
Median (IQR), Range Years Working	11 (15.8), 1–37
n with SCI/D Patients in Caseload ≥ 25%	11

### Professional contribution to MTM

Each profession talked about their contribution to MTM for persons with SCI/D. In general, most participants felt that the care they provided for this population was the same or similar to the care that they provided to other patients, with some differences mostly relating to the physical disabilities associated with SCI/D. Pharmacists described their roles as “looking at the entire patient and making sure from kind of top to bottom that all of their issues are being addressed” (C04). They also described themselves as “the medication experts” (C02) responsible for “drug plan[s]” (C06) and optimizing medication therapies to ensure “medications are safe and effective” (C03) and are “what [patients] need and not more than what they need and are giving them kind of the most benefit” (C01). Pharmacists described conducting medication reviews and a “cognitive [and] physical assessment” (C04) to optimize adherence and ensure medications were “appropriate for administration” (C07) for the person with SCI/D. Pharmacists talked about providing medication counselling and sharing information about the indications, side effects, and potential interactions of medication. They dispensed medications, sometimes in compliance packaging (e.g., blister packages and dosette boxes), and sometimes delivered medications to patients. When medication monitoring was mentioned, it was mostly in the context of monitoring for adherence; however, some pharmacists also discussed monitoring for effectiveness and side effects. Finally, pharmacists also described making recommendations to prescribers about therapeutic alternatives.

Family physicians described two main MTM roles for persons with SCI/D. First, family physicians talked about being the “main prescriber” (C26) which involved many medication-specific activities: prescribing, de-prescribing, modifying therapies, monitoring side effects and complications, assessing indications and contraindications, maximizing adherence, providing education, and following-up with patients. The second contribution was providing “central coordination” (C22), describing their role as “looking at the big picture in making sure…all the pieces are moving in the right direction…” (C15), rather than being an “expert in [SCI/D] medications” (C15). As the central coordinator, family physicians talked about coordinating with specialist physicians about SCI/D-specific medications. Specialist physicians explained that they make “recommendations for medications that are specific to spinal cord injury” (C28) as well as provide support with “emotional coping” (C30) related to living with an SCI/D. In addition to prescribing SCI/D-specific medications, they also discussed prescribing medical devices, harmonizing medications from all prescribers (i.e., assessing drug-drug interactions), and making recommendations to patients about community supports (e.g., community pharmacists, local SCI/D organizations).

Nurses and care coordinators took on a supportive role as health navigators, assisting persons with SCI/D in navigating the healthcare system to access appropriate and physically accessible professionals and community recourses. These providers assisted patients in preparing for appointments with doctors; for example, “writing out what they want to say to the physician” or educating them on “what they should say in order to get their message across” (C12). Similarly, the other rehabilitation professionals also discussed assisting persons with SCI/D in preparing for their physician appointments through teaching self-advocacy skills. They emphasized their role in teaching life skills education, sharing information “about spinal cord injury with them in relation to how to live their daily life” (C14, Occupational Therapist), including establishing routines (e.g., bowel/bladder, medication schedules), and finding accessible services (e.g., pharmacies). Rehabilitation professionals also discussed conducting physical and cognitive assessments to determine medication taking abilities and making referrals to physicians, dieticians, or other healthcare providers as needed. Many participants described overlapping MTM roles with other providers such as tailoring medications, providing medication education, and exploring alternatives to medications. These overlapping roles are described in more detail below.

#### Tailoring medications

Most participants discussed tailoring medications to the needs of each person with SCI/D in a similar manner to how medications are tailored for other patients. For pharmacists and physicians, medication tailoring was most frequently discussed in the context of assessing the therapeutic benefits in comparison to the associated risks (i.e., side effects), and through trial and error to find the most suitable medication. Trial and error of pain medications was especially common for persons with SCI/D.

*Well, often [the] side effects most people get are expected and a normal consequence, like if someone’s on gabapentin for neuropathic pain, and they feel some fatigue within the first few days, I will ask them - I will reassure them that that’s expected and it will likely improve. If it doesn’t, then I’m willing to make a dose adjustment, change medications or stop that therapy altogether.* (C16, Family Physician)

Rehabilitation professionals discussed medication tailoring in terms of incorporating medications into patients’ individual routines (i.e., timing and frequency) and ensuring patients’ functional ability to take medications (i.e., opening medication vials/bottles/blister packages or having access to attendants/caregivers to assist with medication taking), both of which were often more challenging for persons with SCI/D because of their physical limitations.

*They learn how to fine tune their medication schedule and then they also learn how to go, you know, go back to their doctor, go to pharmacist and ask him, you know – I think to - to make sure the frequency or the time of day that they should take the medication is in sync with the new life that they’re learning about themselves, to live in the community.* (C14, Occupational Therapist)

#### Providing education

All participants described providing education to persons with SCI/D concerning medications. Rehabilitation specialists discussed educating patients on how to effectively communicate with physicians and on facilitating medication taking behaviours (e.g., routines). Pharmacists and physicians discussed counselling patients and caregivers on medication indications, anticipated therapeutic benefits, potential side effects, the importance of follow-ups, and medication administration.

*Mainly just through counselling, kind of describing the need for each medication… I guess counselling, providing education around the indication, the importance of the monitoring for side effects and then the need to continuously follow up with the clinic and blood with work.* (C05, Community Pharmacist)

#### Exploring medication alternatives

About half of all participants discussed exploring non-pharmacological treatments to manage symptoms of SCI/D. Such treatments commonly included “massage therapy, physiotherapy, acupuncture” (C01, Community Pharmacist), “psychologist, or… aquatic exercises or electrical stimulation, sometimes even diet” (C02, Community Pharmacist), “exercise…heat, ice” (C28, Specialist Physician), and using adaptive or medical devices. Rehabilitation professionals often discussed alternatives to medications as pain management strategies.

*So, not even just medications based, but things like acupuncture, other kind of techniques that can be done. So, I think that something like pain is a very difficult problem to solve and we should be trying as many different things as we can.* (C21, Physical Therapist)

### MTM barriers and enablers

Five main factors were identified that impacted participants’ abilities to support MTM for persons with SCI/D: a) patient self-management skills, b) provider knowledge and confidence, c) provider-patient relationships, d) interprofessional collaboration, and e) provider funding models including the use of technology-supported consultations. All factors were both barriers and enablers, depending on the presence or absence of the factor. See Table 2 for supporting quotes for each factor and participant recommendations on addressing barriers.

**Table 2:** Barriers and Enablers of MTM for Persons with SCI/D with Participant Quotes and Recommendations

**1. Patient self-management skills including knowledge of SCI/D-specific medications and information seeking behaviors**	
*Maybe it’s the educational level of the person. If it’s really, really, rudimentary level then it’s a little bit – can be a little bit tough, you know, to reinforce certain ideas.* (C08, Community Pharmacist) *…they want to know more and they want to be educated more, but* *sometimes where they get their education, their resources are not, are not appropriate sites*. (C09, Community Pharmacist) *The longer the person has [the] injury, the more insight they have into their body and you have to give credit... I could say once they are, you know, more medically stable, you really need to listen to them and listen to how they want to live their life and how you can complement their life with the medication* *to control the spasms, to control the pain at the right time so that they can have a meaningful life.* (C14, Occupational Therapist) *…one of the ways that individuals with spinal cord injury… cope with a primary care practitioner who may only have one or two people with spinal cord injury in their practice, is* *they become experts in the care of their own disease… individuals with spinal cord injury become very well-educated consumers for the most part and* *they understand their bodies better than anyone*. (C28, Specialist Physician)	• Spend time with patient to: ◦ Share medication-related information ◦ Understand how the therapy will complement/ improve their lives ◦ Identify reliable sources of information
**2. Provider knowledge and confidence with SCI/D secondary health complications and related medications**	
*…because I’m not like primarily focused on the SCI patients, that I don’t really put like a—I don’t know that much about specifics that should be addressed. So,* *I probably don’t provide education that might be more specific or tailored to them* *just because I’m unaware of that information and where to find it.* (C05, Community Pharmacist) *…based on your experiences you know what other medications are commonly used in SCI* *so, yeah, you feel comfortable enough to kind of be like to the doctor maybe we can try this.* (C18, Occupational Therapist) *…you know, recognizing that for* *the average primary care practitioner, they are going to have a very small number of these people* *and so, to expect that they, by themselves, can maintain a level of clinical expertise necessary or appropriate to the complexity or the specifics of the type of health problems and medication issues that spinal cord patient experience, I think that’s not reasonable.* (C22, Family Physician)	• Access to guidelines, best practices, websites, or information sheets on common SCI/D-related medications • Create SCI/D-specific continuing education courses
**3. Relationships and trust between healthcare providers and patients/their caregivers**	
*… I think it also* *comes down to their comfort level with the pharmacist* *that they are speaking with. I found that at times when they don’t know the pharmacist or they’re not familiar with the pharmacist, it might just be the medication and that’s it. Whereas* *if they’re comfortable, they can actually have the conversation with the pharmacist to explain how they’re doing.* (C02, Community Pharmacist) *What I would like most is some sort of feedback mechanism about whether they’re taking their meds or not and why not. And I just – I can ask them but* *the answer to that question is always yes, I take them. You know. Nobody says I don’t. It’s always yes…they want me to like them. I don’t – I think they – they’re concerned about honesty. Just, you know… they don’t want to piss off their doctor… But* *I like when people tell me the truth.* (C15, Family Physician) *Somebody with spinal cord injury, they might be on a medication that was prescribed to them by a specialist who they know they can only see once a year and they really just don’t want to mess with it because they don’t have confidence that whoever their regular is would have the same knowledge in terms of making changes. So, I think just* *the confidence of a person who can provide them with that information is valuable.* (C19, Occupational Therapist)	• Create an inviting environment • Ensure two-way communication • Listen and understand patients' concerns
**4. Interprofessional collaboration through multidisciplinary workplaces and through access to SCI/D specialists for clinical support**	
*I rely on either the specialist provider and reach out to them if I’ve got questions or our local pharmacist* *who can also access other pharmacists, for instance, who might be in particular clinics where they are providing care to a lot of spinal cord patients.* (C22, Family Physician) *…there has to be support in place in the either local community or regionally so that, for instance, the person can access timely medication support information that they would need. They understand enough about medications and interactions,* *what they don’t necessarily have is access to the specifics as they pertain to somebody with spinal cord injury. So, I think that’s the key thing. If they can be—the ideal is like—it works very well in our setting because of the onsite interdisciplinary team* *and we’re fortunate, again, because linked to the hospital and our office design allows the accommodation.* (C22, Family Physician)	• Work in collaborative, multidisciplinary practices • Access knowledgeable specialists • Create an electronic record system that is shared with the patient's circle of care
**5. Community pharmacist and physician funding models**	
*Sometimes, you know, sometimes time is hard, you know, to talk to a patient or be with that patient or just, you know, that touch point.* *It takes time,* *I mean if you ask anybody, everybody will always tell you time…* *you wish you would have more time to follow up to make sure that people are doing things properly.* (C09, Community Pharmacist) *Some* *physicians like to talk about one issue per visit, so we do have a barrier there with physicians of being able to say well, you know, if the transportation to the physician’s office is hugely time consuming and difficult and you have to arrange or pay for a cab or it takes you, you know, you have to book a week, you know, in advance, for the transportation, all those kinds of things. Then* *we would love to have an avenue to have multiple issues dealt with in one appointment, but in fairness to the physician, they can really only bill for one issue* *and one medication per visit. So, and they don’t have any financial ability to really cooperate with a patient with spinal cord injury needs.* (CC01, Care Coordinator) *Yeah, I mean that’s probably a little bit more unique to our practice because we do – do* *some emailing* *and we do – do some* *videoconferencing, but* *that wouldn’t be typical of most practices. […] So, that is done officially I can follow-up on patients. It’s an easier sort of a thing.* *The problem is that you can’t bill for that though, so it’s not – it’s not conducive to most practices…* (C23, Family Physician)	• Change billing models to allow for longer appointment times: ◦ Reimburse pharmacists for more patient care services ◦ Allow discussion of multiple issues during one physician appointment ◦ Account for technology supported consultations
**5.1 Alternative appointment formats**	
*I’m sort of* *more flexible on doing phone appointments…* (C15, Family Physician) *…we have* *support through PCVC (personal computer video conferencing) support that they don’t have to necessarily come in in-person or virtual visits…* (C17, Family Physician) *Quite often what you would do is just,* *you’d book an afternoon which is just strictly home visits…I can* *do telephone follow up… and part of the reason for that is just cuz of the challenges for the person to be able to come to the office easily.* (C22, Family Physician)	• Use technology supported consultations: ◦ Video-conferences ◦ Telephone • Offer home visits

*Abbreviations*: *MTM* Medication therapy management, *SCI/D* Spinal cord injury/dysfunction

Barriers and enablers are set in bold

#### Patient self-management skills

Most participants discussed patient knowledge of their SCI/D-specific medications and patient information-seeking behaviours as both barriers and enablers for the provision of MTM. Many different providers explained that some patients with SCI/D had cognitive impairments or limited cognitive abilities, a barrier to supporting MTM. In comparison, the specialist physicians described persons with SCI/D as often becoming “experts in the care of their own disease” (C28), which was viewed as an enabling factor to the provision of MTM.

Patient information-seeking behaviours were also considered to be both barriers and enabers depending on the source. For example, pharmacists and physicians described information from “Google God” (C09, Community Pharmacist) and “Doctor Google” (C26, Family Physician) as a barrier when it contradicted information that they were sharing with the patient. To overcome this barrier, participants described providing reliable websites and other sources of information to persons with SCI/D.

#### Provider knowledge and confidence

Provider knowledge and confidence were both barriers and enablers depending on their experience working with the population. Overall, most pharmacists desired more knowledge about the SCI/D condition, common complications, and common medications. Most pharmacists attributed their lack of knowledge to the small number of persons with SCI/D in their caseloads. The desire for more knowledge influenced pharmacists’ confidence when sharing information with persons with SCI/D, as a community pharmacist explained, “I think in terms of just for confidence, you never want to tell a patient something wrong” (C02). Some pharmacists felt that their desire for more knowledge was hampered by the fact that clinical drug trials “don’t usually include patients with spinal cord injuries. So, it’s hard to know if there needs to be any adjustments made for them because the data is not there” (C04, Hospital and Community Pharmacist).

The rehabilitation specialists as well as the family and specialist physicians all felt that they had much more SCI/D-specific knowledge and therefore, more confidence giving recommendations on medication initiation, access, and use. These participants generally had more persons with SCI/D in their caseloads. Despite the high levels of knowledge and confidence reported by these participants, some felt that the “average primary care practitioner” (C22, Family Physician) may not be as knowledgeable and confident with this patient population, nor should they be expected to be, because most see very few persons with SCI/D in their clinical practices.

#### Provider-patient relationships

All providers spoke about the importance of a holistic patient-centred approach, which was facilitated by positive provider-patient relationships. As a community pharmacist described, establishing a level of comfort through an inviting and open environment made it easier for patients to discuss experiences with medications. Developing rapport and trust with their patients was identified as a key enabler when supporting persons with SCI/D to manage medications.

The importance of good provider-patient relationships and trust was discussed in detail by many participants in the context of medication deprescribing and therapeutic substitutions. These participants explained how open communication through providing unbiased information and listening to the patient, contributed to a good relationship and increased patient buy-in to the medication treatment plan. A family physician explained that persons with SCI/D “like to be involved in the decision making and I think if you involve them, they are actually probably more likely to accept the therapy and be adherent…” (C26). Relationships and trust also extended to family caregivers of persons with SCI/D, as a family physician explained, “I make an effort that their guest [i.e., caregiver] also understands the information, to get both of them on board with whatever therapy…” (C15).

#### Interprofessional collaboration

Participants described interprofessional collaboration, either through multidisciplinary team-based environments or through communications with an external SCI/D specialist, as a critical enabler to supporting MTM. Interprofessional collaboration was identified as an enabler by all professions. All participants who worked in a team-based environment spoke highly of the care they were able to provide to their patients with SCI/D. For example, a family physician in a multidisciplinary primary care team spoke about his ability to bring a pharmacist or a nurse into appointments to get second opinions or advice. Co-location of multi-disciplinary professionals was described as an enabler of MTM, as providers in these settings described feeling more supported when providing care to someone with SCI/D. Participants who did not work in a multidisciplinary team spoke of the need to consult with other healthcare professionals for clinical support; however, knowledge around which professionals could act as external clinical supports and how to access them was sometimes lacking. Rehabilitation specialists discussed relaying medication adherence concerns to physicians and pharmacists, while family and specialist physicians discussed relying on pharmacists for medication interactions and suggestions for therapeutic alternatives. As medication experts, some community pharmacists expressed a desire to be more involved in the initiation of prescription medications for persons with SCI/D, instead of providing feedback to the prescriber when dispensing new medications.

#### Pharmacist and physician funding models

Current funding models were identified as a barrier to the provision of patient-centered care and optimal MTM. Specifically, participants talked about the funding model of pharmacists (i.e., heavily product-based compensation) and physicians (i.e., fee for service, one issue per appointment). For example, despite pharmacists’ expanded scope of practice, which now includes more services (e.g., initiating, adapting and renewing prescriptions, administering substances by injection or inhalation [27]), some pharmacists explained that encounters with complex patients take more time, which is not always feasible in their fast-paced environment. One community pharmacist also explained that despite the expanded scope, “a lot of community pharmacists, they do limit themselves to, I just want to fill out scripts and that’s it” (C02).

Many participants discussed the limitations on physician appointments due to physician billing models. Specifically, participants explained that time was limited, and “physicians [do] not have the ability to book or bill for longer appointments that would suit the needs of a person with a spinal cord injury” (CC01, Care Coordinator); therefore, often only one issue could be discussed per visit. Physicians also commented on this limitation saying that, “if physicians were actually compensated properly to spend the time that all these patients warrant” (C26, Family Physician), then they would be able to better search for relevant SCI/D medication information and collaborate with other professions on behalf of the patient. This concern was described as problematic for persons with SCI/D because of the difficulties these individuals often have in physically accessing the physician’s office (e.g., scheduling appointments, arranging transportation). Technology-supported consultations were suggested as alternatives to in-person consultations by rehabilitation specialists. Despite limited compensation for providing services by telephone or video-conferencing, these alternative appointment formats were identified as an enabler for MTM by community pharmacists and physicians, who described conducting follow-up telephone calls and providing home visit services. Physicians also described using video-conferencing with persons with SCI/D, when possible, to avoid unnecessary trips to the clinic.

Participants recommended multiple strategies for improving MTM for persons with SCI/D, which included increasing knowledge of SCI-specific medication for both patients and providers, improving relationships and communication between providers and between providers and persons with SCI/D, and changing funding models of physicians and pharmacists (see Table 2).

### Summary

Participants discussed their experiences and roles supporting MTM among persons with SCI/D. When comparing care for individuals with SCI/D, participants overwhelmingly felt that the care they provided was the same or similar to that provided to other patients. For example, a pharmacist explained that “the strategies are the same, the problems may be different” (C04) and occupational therapists said “the nature of the work is the same” (C14) and “I’m always going to provide client-centered care” (C18). Despite the similar care provided, some SCI/D-specific differences were discussed including patients’ physical limitations (e.g., administering mediations, accessing services, requiring special equipment) and increased patient medical complexity requiring more clinical time. For example, a family physician explained:

*…practically speaking, it’s – they are much, much longer appointments. You need a much bigger space. You need to schedule and coordinate appointments with respect for their transportation to and from the office, their attendant support, their home bowel regimen, their urinary continence issues, their funding, There’s a lot.* (C16, Family Physician)

## Discussion

To our knowledge, this is one of the first qualitative studies to explore the experiences of healthcare and service providers with MTM for persons with SCI/D, as well as the perceived barriers and enablers of optimal MTM. Overall, participants described their role in MTM for SCI/D to be similar to other patient populations; however, several nuances were identified when providing clinical care to persons with SCI/D due to their medical complexities. Five main factors were identified in this study that influenced healthcare and service providers’ ability to support MTM for persons with SCI/D: patient self-management skills, provider knowledge and confidence, provider-patient relationships, interprofessional collaboration, and provider funding models.

Interestingly, few of the barriers and enablers identified by healthcare and service providers are specific to the SCI/D population, but rather seem to be amplified due to the medical complexity and disability associated with the condition. For example, participants discussed physicians’ general lack of time and an inability to discuss more than one issue as barriers limiting MTM for persons with SCI/D that resulted from the constraints of current funding models (e.g., fee-for-service). These time constraints have been noted previously when providing care for persons with multimorbidity and disability [28, 29]. Most participants agreed that appointments for persons with SCI/D generally required more time and would be more effective if multiple issues could be discussed during the same visit. This was especially important for persons with SCI/D due to the barriers that individuals experience in scheduling and attending physician appointments – barriers which are supported by recent literature on primary care access for both traumatic spinal cord injury [30] and individuals who use wheelchairs [31].

The additional funding for longer appointments for patients with complex needs would also enable more attention to fostering positive provider-patient relationships. For example, salaried physicians in some primary care models serving vulnerable and complex patient populations (e.g., community health centres in Ontario) have more time for longer appointments to build positive patient-provider relationships [32–34]. These therapeutic relationships are especially important for common SCI/D concerns such as pain management, and have been associated with patient-centered care, provider empathy, open communication, and trust [35, 36]. Previous research has shown the importance of positive relationships for self-management in general [37, 38] and more specifically for medication adherence [39, 40].

Specific to SCI/D, our study identified that a key factor was provider knowledge and confidence with MTM for this population. As medication experts, some pharmacists expressed a desire to be more involved in initiating medications for persons with SCI/D. These pharmacists felt that they had the necessary skill set to provide valuable input to prescribers. This was especially salient given that some family physicians did not feel it was their role to have specialized medication knowledge relating to common SCI/D secondary conditions. Previous research has identified a similar concern among primary care physicians [28, 41]. McMillan and colleagues identified that while physicians acknowledge important knowledge gaps and the need for additional general education about providing care for persons with mobility issues, the lack of time and substantial effort required were barriers [41]. To address some of these barriers, enhancing interprofessional collaborations between pharmacists and physicians may provide more clinical support for complex MTM. Recent studies in varying jurisdictions have found that collaborative physician-pharmacist prescribing for a variety of conditions have been well-received by patients [42], have resulted in improved treatment, cost savings, and improved quality of life [43], and, for prescription renewals, have resulted inmore identified and corrected medication-related problems [44]. These studies demonstrate the benefit of improving collaboration during prescribing processes, which may also be applicable for persons with SCI/D.

Finally, participants in our study highlighted the importance of patient self-management skills as a factor influencing their ability to support MTM. In particular, rehabilitation specialists spoke about preparing persons with SCI/D for physician visits by teaching self-advocacy and communication skills to overcome short appointment times. Creating opportunities for open dialogue so persons with SCI/D are less rushed during clinical encounters would ideally encourage concerns to be ‘heard’ by their providers [30] and for patients to be treated with empathy [45]. Further, in our study, rehabilitation specialists described co-creating written lists of topics to discuss (e.g., symptoms, medication concerns) to empower patients for their future clinical visits. Similarly, a recent qualitative study conducted in the United States found that patients with diabetes and physicians valued having a list of key priorities created in advance of appointments [46]. Relevant themes identified included the importance of prioritizing discussion topics in advance of appointments, challenges associated with appointment time constraints, and the need for strategies to help patients prepare for physician visits [46]. In our study, we found that rehabilitation specialists played a crucial role in addressing these strategies for persons with SCI/D.

### Study limitations

This study had a few limitations. While substantial efforts were made to interview a diverse range of healthcare providers, input from nurses and specialist physicians (e.g., urologists, physiatrists, etc.) was limited. Because of our snowball sampling strategy, our family physician participants likely had more clinical expertise with SCI/D compared to most family physicians practising in Canada.

### Recommendations for future research

Future research should include perspectives of family physicians that are less experienced with SCI/D and have more input from nurses and specialist physicians. It is possible that less experienced clinicians may identify additional or different factors that impact their ability to support MTM. Future research might also explore healthcare providers’ perceptions and experiences in supporting MTM for other populations with complex needs to identify commonalities of barriers and enablers. Further, it would be of interest to understand the perceptions and experiences of persons with SCI/D about MTM and how these compare with those of healthcare and service providers.

## Conclusion

Overall, healthcare and service providers described the care they provided to persons with SCI/D as similar to the care they provided to other patients, with differences resulting mostly from the physical limitations and medical complexities of persons with SCI/D. Each profession had distinct views on their roles in supporting MTM for persons with SCI/D with some overlap in roles. The barriers and enablers of MTM identified may contribute to strategies to improve medication management support for persons with SCI/D.

## Data Availability

The datasets used and/or analysed during the current study are not available due to protecting participants’ anonymity.

## Abbreviations

MTM: Medication therapy management
SCI/D: Spinal cord injury/dysfunction
